# Rolling Bearing Fault Diagnosis Based on Successive Variational Mode Decomposition and the EP Index

**DOI:** 10.3390/s22103889

**Published:** 2022-05-20

**Authors:** Yuanjing Guo, Youdong Yang, Shaofei Jiang, Xiaohang Jin, Yanding Wei

**Affiliations:** 1 Zhijiang College, Zhejiang University of Technology, Shaoxing 312030, China; guozi@zzjc.edu.cn; 2 College of Mechanical Engineering, Zhejiang University of Technology, Hangzhou 310023, China; xhjin@zjut.edu.cn; 3 Key Laboratory of Advanced Manufacturing Technology of Zhejiang Province, College of Mechanical Engineering, Zhejiang University, Hangzhou 310027, China; weiyd@zju.edu.cn

**Keywords:** rolling bearing, fault diagnosis, successive variational mode decomposition, squared envelope spectrum, EP index

## Abstract

Rolling bearing is an important part guaranteeing the normal operation of rotating machinery, which is also prone to various damages due to severe running conditions. However, it is usually difficult to extract the weak fault characteristic information from rolling bearing vibration signals and to realize a rolling bearing fault diagnosis. Hence, this paper offers a rolling bearing fault diagnosis method based on successive variational mode decomposition (SVMD) and the energy concentration and position accuracy (EP) index. Since SVMD decomposes a vibration signal of a rolling bearing into a number of modes, it is difficult to select the target mode with the ideal fault characteristic information. Comprehensively considering the energy concentration degree and frequency position accuracy of the fault characteristic component, the EP index is proposed to indicate the target mode. As the balancing parameter is crucial to the performance of SVMD and must be set properly, the line search method guided by the EP index is introduced to determine an optimal value for the balancing parameter of SVMD. The simulation and experiment results demonstrate that the proposed SVMD method is effective for rolling bearing fault diagnosis and superior to the variational mode decomposition (VMD) method.

## 1. Introduction

Rolling bearing is an important part widely used in rotating machinery, such as wind turbines [1], high-speed railways [2], helicopters [3] and electric vehicles [4]. As rolling bearing always operates under harsh working conditions, ranging from high speed and alternating speed to heavy load and alternating load, its inner race, outer race, and balls are prone to suffer from various kinds of damages, including fatigue pitting, wear, spalling, cracking, etc. Hence, it is of great significance to take effective technical measures for rolling bearing health condition monitoring and fault diagnosis to guarantee the reliable operation and long-term economic benefits of rotating machinery. When a localized defect occurs in the inner raceway, outer raceway, or balls of a rolling bearing, a series of impact events are excited because the damaged rolling contact surface lacks smooth support [5]. Specifically, for a rolling bearing under steady working conditions, these impact events are presented as periodic impulse features in the vibration signal. Different impulse feature frequencies are closely related to the fault characteristic frequencies (FCFs) of different components of a rolling bearing. Therefore, it comes very naturally to extract the fault characteristic frequency (FCF) from sampled vibration signal and then diagnose a rolling bearing fault. However, as a rolling bearing is usually installed inside the machinery together with other rotating parts, the impulsive vibration features excited by fault damage will attenuate along the transfer path from the source to the location of the vibration sensor. Based on this, as well as taking into consideration background noise and interference components caused by other parts, the weak fault features’ extraction from a vibration signal for rolling bearing fault diagnosis is usually a challenging task and has attracted considerable attention. Thus, many useful methods have been developed.

One of the most effective and practical methods is signal decomposition, including wavelet packet decomposition [6], empirical mode decomposition (EMD) [7,8], local mean decomposition (LMD) [9,10], empirical wavelet transform (EWT) [11,12,13,14], variational mode decomposition (VMD) [15,16,17,18], and so on. Signal decomposition can extract the useful component containing fault characteristic information and thus achieve the purpose of removing noise and interference components. Among the aforementioned signal decomposition methods, VMD decomposes a signal into an ensemble of band-limited sub-signals called modes [19], and it currently receives extensive study and application due to its complete mathematical principles and ability to avoid the shortcomings of sensitivity to noise, end effects, and mode mixing, which are inherent in EMD and LMD [20,21,22,23]. Nevertheless, there are two critical parameters affecting the performance of VMD that need to be pre-set properly for VMD implementation: the balancing parameter and number of modes. Although these two parameters can be directly pre-set by experience or experiments, the method has the drawback of blindness and is difficult to obtain excellent performance of VMD. Consequently, researchers usually utilize some intelligent optimization algorithms to determine the values of the two parameters, such as the genetic algorithm [24,25], particle swarm optimization [16,26], differential search algorithm [27], Archimedes optimization algorithm [28], grey wolf optimization [29,30], whale optimization algorithm [31], cuckoo search algorithm [32], sparrow search algorithm [33], and so on.

Although these intelligent optimization algorithms have achieved successful applications for the determination of VMD parameters, there are still some drawbacks that cannot be ignored. The implementation of an intelligent optimization algorithm usually requires a lot of initializations and iterative calculations, which is a highly time-consuming process. In addition, the intelligent optimization algorithm can easily fall into the local optimal value or even be difficult for convergence. Therefore, finding the optimal values for the two parameters of VMD using an intelligent optimization algorithm still needs further study.

As an improvement of VMD, a novel signal decomposition method known as variational mode extraction (VME) was proposed by Nazari and Sakhaei [34]. VME is homologous with VMD but decomposes a signal into two modes, the desired mode and residual signal, which avoids the trouble of determining the modes number associated with VMD. However, in the application of a rolling bearing fault diagnosis, it is often difficult to determine the initial center frequency and also not easy to optimize the balancing parameter for VME [35]. Based on this, Nazari and Sakhaei proposed an efficient and fast adaptive signal decomposition method named Successive-VMD (SVMD) [36]. In essence, the implementation of SVMD is done by successively applying VME on a signal. SVMD not only avoids the need to know the number of modes and has lower computational complexity in contrast to VMD, but also skirts the problem of initial center frequency determination in VME. Nevertheless, SVMD is confounded by the basic trouble of difficulty optimizing the balancing parameter. Different balancing parameter values would likely lead to different numbers of modes. Moreover, how to select the useful mode containing fault characteristic information from the multiple modes obtained by SVMD also remains an important issue needing further study.

Based on the above analysis, this paper proposes a rolling bearing fault diagnosis method based on SVMD, in which the target mode containing ideal fault characteristic information is selected from the modes using a novel index, named the energy concentration and position accuracy (EP) index. Accordingly, the line search method is adopted to achieve the globally optimal value for the balancing parameter of SVMD. The subsequent sections of this paper are organized as follows. The SVMD algorithm is briefly introduced in Section 2. Section 3 explains the principle of the proposed EP index. In Section 4, the line search method for the optimal value of the balancing parameter and the corresponding SVMD method for rolling bearing fault diagnosis are summarized. The simulation signal analysis using the SVMD method and the EP index is described in Section 5. Three datasets associated with three kinds of rolling bearing faults are used to verify the effectiveness of the proposed method in Section 6. Concluding remarks are presented in Section 7.

## 2. Successive Variational Mode Decomposition

Essentially, the SVMD of a signal *x*(*t*) requires successively performing VME on the signal *x*(*t*) until all modes are extracted, or the reconstruction error, defined as the error between the original signal *x*(*t*) and sum of the modes, is less than a given threshold [36]. In order to achieve the SVMD method, the original signal *x*(*t*) is first assumed to be decomposed into two signals, i.e., the *L*th mode $u_{L}(t)$ and residual signal $x_{r}(t)$:

(1) $$x(t)=u_{L}(t)+x_{r}(t)\;$$

where the residual signal $x_{r}(t)$ is also composed of two parts, i.e., the sum of the previously obtained modes and the unprocessed part $x_{u}(t)$ of the original signal *x*(*t*):

(2) $$x_{r}(t)=\sum_{i=1}^{L-1}u_{i}(t)+x_{u}(t)\;$$

The SVMD method for the *L*th mode $u_{L}(t)$ extraction is established based on four criteria, which are briefly described as follows [36]: (1) Each mode should be compact around its center frequency; (2) the spectral overlap between $u_{L}(t)$ and $x_{r}(t)$ should be minimized; (3) the energy of $u_{L}(t)$ at frequencies around the center frequencies of the previously obtained modes should also be minimized; (4) the original signal *x*(*t*) should be completely reconstructed from the *L* modes and $x_{u}(t)$. Hence, when the *L* − 1 modes are known, the task of the *L*th mode extraction can be transformed into a constrained minimization problem, as follows:

(3) $$\begin{array}{l} \underset{u_{L},\omega_{L},x_{r}}{\min}\left\{\alpha {\left\|\partial_{t}\left[\left(\delta (t)+\frac{j}{\pi t}\right)*u_{L}(t)\right]e^{-j\omega_{L}t}\right\|}_{2}^{2}+{\left\|\beta_{L}(t)*x_{r}(t)\right\|}_{2}^{2}+\sum_{i=1}^{L-1}{\left\|\beta_{i}(t)*u_{L}(t)\right\|}_{2}^{2}\right\}\\ \mathrm{subject}\;\mathrm{to}:u_{L}(t)+x_{r}(t)=x(t) \end{array}$$

where $\omega_{L}$ is the center frequency of the *L*th mode, *α* is a parameter for balancing, ∂*t* denotes the partial derivative with time *t*, *δ*(*t*) is the Dirac function, and * is the convolution operator. *β*(*t*) is the impulse response of the filter $\hat{\beta }(\omega )$ used to filter the frequencies in $x_{r}(t)$ overlapping with $u_{L}(t)$ to satisfy criterion (2). The filter $\hat{\beta }(\omega )$ can be expressed as:

(4) $$\hat{\beta }(\omega )=\frac{1}{\alpha {\left(\omega -\omega_{L}\right)}^{2}}\;$$

where $\beta_{i}(t)$ is the impulse response of the filter ${\hat{\beta }}_{i}(\omega )$ used to filter the frequencies in $u_{i}(t)$ overlapping with $u_{L}(t)$ to satisfy criterion (3). The filter ${\hat{\beta }}_{i}(\omega )$ can be expressed as:

(5) $${\hat{\beta }}_{i}(\omega )=\frac{1}{\alpha {\left(\omega -\omega_{i}\right)}^{2}},i=1,2,\cdot \cdot \cdot ,L-1\;$$

To convert the constrained minimization problem described in Equation (3) into an unconstrained optimization problem, the quadratic penalty term and Lagrangian multiplier *λ* are jointly introduced to establish the augmented Lagrangian function, as follows:

(6) $$\begin{array}{cc} L(u_{L},\omega_{L},\lambda )= & \alpha {\left\|\partial_{t}\left[\left(\delta (t)+\frac{j}{\pi t}\right)*u_{L}(t)\right]e^{-j\omega_{L}t}\right\|}_{2}^{2}+{\left\|\beta_{L}(t)*x_{r}(t)\right\|}_{2}^{2}+\sum_{i=1}^{L-1}{\left\|\beta_{i}(t)*u_{L}(t)\right\|}_{2}^{2}\\ & +{\left\|x(t)-\left(u_{L}(t)+\sum_{i=1}^{L-1}u_{i}(t)+x_{u}(t)\right)\right\|}_{2}^{2}+\langle \lambda (t),x(t)-\left(u_{L}(t)+\sum_{i=1}^{L-1}u_{i}(t)+x_{u}(t)\right)\rangle \end{array}$$

According to the Parseval’s theorem, Equation (6) can be converted to the frequency domain form and be rewritten as:

(7) $$\begin{array}{cc} L\left(u_{L},\omega_{L},\lambda \right)= & \alpha {\left\|j(\omega -\omega_{L})\left[\left(1+\mathrm{sgn}(\omega )\right)\cdot {\hat{u}}_{L}(\omega )\right]\right\|}_{2}^{2}+{\left\|{\hat{\beta }}_{L}(\omega )\cdot \left({\hat{x}}_{u}(\omega )+\sum_{i=1}^{L-1}{\hat{u}}_{i}(\omega )\right)\right\|}_{2}^{2}+\sum_{i=1}^{L-1}{\left\|{\hat{\beta }}_{i}(\omega )\cdot {\hat{u}}_{L}(\omega )\right\|}_{2}^{2}\\ & +{\left\|\hat{x}(\omega )-\left({\hat{u}}_{L}(\omega )+{\hat{x}}_{u}(\omega )+\sum_{i=1}^{L-1}{\hat{u}}_{i}(\omega )\right)\right\|}_{2}^{2}+\langle \hat{\lambda }(\omega ),\hat{x}(\omega )-\left({\hat{u}}_{L}(\omega )+{\hat{x}}_{u}(\omega )+\sum_{i=1}^{L-1}{\hat{u}}_{i}(\omega )\right)\rangle \end{array}$$

As in the VMD and VME methods, the alternate direction method of multipliers (ADMM) algorithm is also used to iteratively solve the above minimization problem, and the specific solution process can be seen in Reference [36]. The final iteratively updating equations of ${\hat{u}}_{L}(\omega )$, $\omega_{L}$, and $\hat{\lambda }(\omega )$ are given as follows:

(8) $${\hat{u}}_{L}^{n+1}(\omega )=\frac{\hat{x}(\omega )+\alpha^{2}{\left(\omega -\omega_{L}^{n}\right)}^{4}\cdot {\hat{u}}_{L}^{n}(\omega )+\frac{\hat{\lambda }(\omega )}{2}}{\left[1+\alpha^{2}{\left(\omega -\omega_{L}^{n}\right)}^{4}\right]\cdot \left[1+2\alpha {\left(\omega -\omega_{L}^{n}\right)}^{2}+\sum_{i=1}^{L-1}\frac{1}{\alpha^{2}{\left(\omega -\omega_{i}^{n}\right)}^{4}}\right]}\;$$

(9) $$\omega_{L}^{n+1}=\frac{\int_{0}^{\infty }\omega {\left|{\hat{u}}_{L}^{n+1}(\omega )\right|}^{2}d\omega }{\int_{0}^{\infty }{\left|{\hat{u}}_{L}^{n+1}(\omega )\right|}^{2}d\omega }$$

(10) $${\hat{\lambda }}^{n+1}={\hat{\lambda }}^{n}+\tau \left[\hat{x}(\omega )-\left({\hat{u}}_{L}^{n+1}(\omega )+\frac{\alpha^{2}{\left(\omega -\omega_{L}^{n+1}\right)}^{4}\left(\hat{x}(\omega )-{\hat{u}}_{L}^{n+1}(\omega )-\sum_{i=1}^{L-1}{\hat{u}}_{i}(\omega )\right)-\sum_{i=1}^{L-1}{\hat{u}}_{i}(\omega )}{1+\alpha^{2}{\left(\omega -\omega_{L}^{n+1}\right)}^{4}}+\sum_{i=1}^{L-1}{\hat{u}}_{i}^{n+1}(\omega )\right)\right]$$

where $\hat{x}(\omega )$ represents the Fourier transform of the original signal *x*(*t*), ${\hat{u}}_{L}^{n}(\omega )$ represents the Fourier transform of the *L*th mode $u_{L}^{n}(t)$ in the *n*th iteration with the center frequency $\omega_{L}^{n}$, *n* is the number of iterations, and τ is the iteration step length. Accordingly, the complete algorithm for SVMD is described in Algorithm 1 [36].
**Algorithm 1.** SVMD Input *x*(*t*) Set *α*, $\varepsilon_{1}$, $\varepsilon_{2}$ and *σ*^2^ Initialize, *L*←0 **repeat**   *L*
←
*
L
*
+ 1
   Initialize ${\hat{u}}_{L}^{1}$, ${\hat{\lambda }}^{1}$, $\omega_{L}^{1}$, *L*←0   **repeat**
     *n*
←
*
n
*
+ 1
       (1) Update ${\hat{u}}_{L}$ according to Equation (8) for all ω≥0
       (2) Update $\omega_{L}$ according to Equation (9)       (3) Update $\hat{\lambda }$ according to Equation (10) using Dual Ascent method for all ω≥0
   **until** convergence: $\frac{{\left\|{\hat{u}}_{L}^{n+1}(\omega )-{\hat{u}}_{L}^{n}(\omega )\right\|}_{2}^{2}}{{\left\|{\hat{u}}_{L}^{n}(\omega )\right\|}_{2}^{2}}<\varepsilon_{1}$
  **until** convergence: $\left|\sigma^{2}-\frac{1}{T}{\left\|x(t)-\sum_{i=1}^{L}u(t)\right\|}_{2}^{2}\right|/\sigma^{2}<\varepsilon_{2}$



Based on the process of Algorithm 1, SVMD can be considered as the solution of *K* optimization problems or the solutions of *K* one-dimensional optimization problems at each frequency, and thus has a lower computational complexity than VMD, which is a solution of the *K*-dimensional optimization problem [36]. Such a superiority of SVMD over VMD was verified in Reference [36]. During the implementation of SVMD, the update parameter *τ* is often set as zero to accelerate the algorithm convergence. The values of the convergence tolerance, ε_1_ and ε_2_, can be set to small positive values in accordance with different requirements. *σ*^2^ is an approximate value of the additive white noise power in the original signal *x*(*t*), which can be estimated using some filters, such as the Savitzky–Golay filter. The most important parameter in the SVMD algorithm is the balancing parameter *α*. A small *α* value may cause the mode mixing problems [36]. For a rolling bearing fault feature extraction, mode mixing means the fault characteristic mode may be seriously interfered by other components or noise. However, if the *α* value is too high, a lot of modes may be generated, most of which are noise or interference components, increasing the difficulty of the useful target mode selection, and the algorithm convergence may be affected. Therefore, the *α* value determination for SVMD is a very important but challenging task, as the proper *α* value usually varies in a large range for different signals. In addition, as there are a number of modes obtained by SVMD, it is often difficult to select the target mode containing the ideal fault characteristic information for a rolling bearing fault diagnosis.

In view of the existing shortcomings in SVMD, we propose a novel index named as the EP index to evaluate the modes obtained by SVMD and accordingly take the line search method to achieve the optimal value of the balancing parameter. Based on these studies, we propose a rolling bearing fault diagnosis method and use a simulated vibration signal of a faulty rolling bearing and three experimental vibration datasets from a rolling bearing testbed to evaluate the performance of the proposed method.

## 3. The EP Index

In this section, we propose the EP index to evaluate the modes obtained by SVMD and indicate the target mode among the modes. The EP index is based on the squared envelope spectrum (SES) analysis of each mode, and its principle is described in detail as follows.

### 3.1. Squared Envelope Spectrum

The purpose of bearing vibration signal processing is to extract the fault features, the most important of which is the FCF. An efficient and direct method for FCF extraction from a bearing vibration signal is the SES analysis. Given a vibration signal *x*(*t*) of a rolling bearing, its SES calculation mainly includes three steps [37].

Step 1: Filter *x*(*t*) around a resonance frequency to remove noise and highlight the structural natural vibration characteristics caused by the impact excitation of bearing damage, and the band-pass filtered signal is expressed as $x_{f}(t)$.

Step 2: Calculate the squared absolute value of the analytic signal of $x_{f}(t)$ to obtain the squared envelope (SE) signal:

(11) $$SE(t)={\left|x_{f}(t)+j\cdot H\left[x_{f}(t)\right]\right|}^{2}\;$$

where H(⋅) denotes the Hilbert transform.

Step 3: Calculate the squared absolute value of the Fourier transform (FT) of the squared envelope signal *SE*(*t*) to obtain the SES:

(12) $$SES(f)={\left|FT\left[SE(t)\right]\right|}^{2}\;$$

where *FT*(·) denotes the Fourier transform.

### 3.2. The EP Index

After a vibration signal of rolling bearing is processed by SVMD, how to evaluate the performance of each extracted mode is of great significance, which has important influence on the value determination of the key parameter α in SVMD and the useful target mode selection. As a rolling bearing mainly consists of an inner race, outer race, and balls, which are prone to damage, we focus on the three corresponding FCFs, i.e., the inner race FCF (*f*_ir_), the outer race FCF (*f*_or_), and the ball FCF (*f*_ba_), of which the theoretical values of a given rolling bearing can be calculated directly. For a mode extracted by SVMD, if it is the expected target mode that contains complete and pure fault characteristics, its SES should satisfy two conditions, described as follows.

(1) Energy concentration: The energy of the SES should concentrate around one of the rolling bearing FCFs as much as possible, and the energy at other frequencies should be as little as possible. Hence, in the SES of the target mode, the amplitude corresponding to the possible FCF should be maximal, whereas the amplitudes at other frequencies should be very low. Accordingly, we propose an index to evaluate the energy concentration of the possible FCF component in the SES of a mode. This index is named the energy concentration (EC) index and calculated with the following two steps.

Step 1: Normalize the SES amplitudes using the following equation:

(13) $$NS(n)=\frac{SES(n)}{\max\left[SES(n)\right]}\;$$

where *SES*(*n*) (*n* = 1, 2, …, *N*) is the discrete form of *SES*(*f*), and *N* is the number of frequency points.

Step 2: Sort the amplitudes of *NS*(*n*) in descending order to obtain the amplitude sequence *SNS*(*n*) and calculate the average value of the differences between the first amplitude and the following *K* amplitudes to obtain the EC index:

(14) $$EC=\frac{1}{K}\sum_{k=1}^{K}\left[SNS(1)-SNS(1+k)\right]\;$$

where, in this paper, the value of *K* is uniformly set as 10. According to the calculation process, 0≤EC≤1 and the EC index of the target mode should be maximal.

(2) Position accuracy: In the SES of the target mode, the frequency corresponding to the maximum energy should be one of the rolling bearing FCFs. It also means that the deviation between the frequency corresponding to the maximum amplitude of the SES and one of the rolling bearing FCFs should be minimized. Consequently, we also propose an index to evaluate the position accuracy of the maximum amplitude of the SES. This index is named the position accuracy (PA) index and calculated using the following two steps.

Step 1: Find the frequency value corresponding to the maximum amplitude of the SES, which is expressed as *f*_ma_.

Step 2: Calculate the PA index as follows:

(15) $$PA={\left(\prod_{m=1}^{M}\left|f_{\mathrm{ma}}-f_{m}\right|\right)}^{1/M}\;$$

where, in this paper, *f_m_* (*m* = 1, 2, …, *M*) are the FCFs of the inner race, outer race, and ball of the rolling bearing, and thus, *M* = 3, *f*_1_ = *f*_ir_, *f*_2_ = *f*_or_, *f*_3_ = *f*_ba_. When one of the three components of the rolling bearing fails, its FCF extracted from the vibration signal will make the PA index close to or equal to zero.

Combining the EC index with the PA index, we propose a new comprehensive index to evaluate the rolling bearing-related fault characteristic information in the extracted modes by SVMD. This index is named the energy concentration and position accuracy (EP) index and defined as follows:

(16) $$EP=\frac{1}{EC^{p}}+\beta \cdot PA\;$$

where *p* is the adjustment coefficient and *β* is the balancing coefficient. Actually, the value of the PA index is several orders of magnitude larger than the value of the EC index and, thus, in order to compensate for this numerical gap, *β* can be calculated directly as follows:

(17) $$\beta =\frac{EC}{EC+PA}\;$$

With regard to the inner race or outer race damage of the rolling bearing, the corresponding FCF represented in the SES of the target mode is usually lightly interfered by other frequency components and can achieve a stable value close to the theoretical value. There is no need to make too many adjustments between the EC and PA index, hence, *p* = 1. However, due to the effect of random slippage of the balls, the FCF of balls is difficult to maintain as a stable value and may deviate greatly from the theoretical value. In this situation, there should be a larger weight for the PA index. In other words, the weight for the EC index should be shrunk, and *p* should be taken a positive value less than 1, such as *p* = 0.5. For the expected target mode extracted from a rolling bearing vibration signal, it should contain complete and sufficient fault characteristic information, and the EP index value of its SES should be minimized. Based on the above analysis, the EP index can be used to optimize the balancing parameter *α* for SVMD and to select the target mode from the results of SVMD.

## 4. The Rolling Bearing Fault Diagnosis Method Based on SVMD and the EP Index

**Figure 1:**
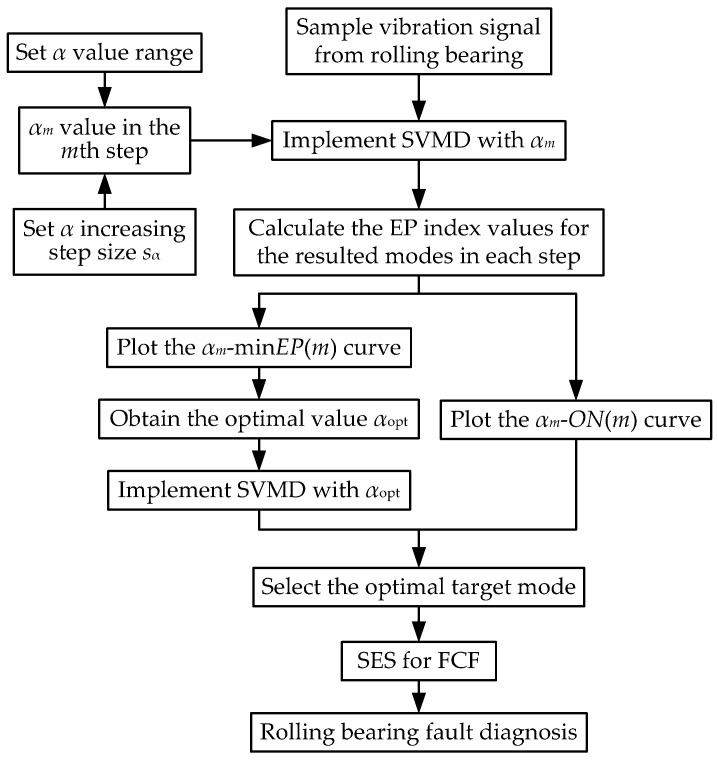
Process flow of rolling bearing fault diagnosis based on SVMD and the EP index.

Considering the SVMD algorithm principle and the vibration signal characteristics of a faulty rolling bearing, this paper proposes a rolling bearing fault diagnosis method based on SVMD combined with the EP index. In the original SVMD method, there is actually only one key parameter, i.e., the balancing parameter *α*. Therefore, for simplicity, the line search method is directly used to obtain a globally optimal value for the balancing parameter *α* in a given range. Since the SVMD algorithm is not very sensitive to *α* as long as the value of *α* varies within a narrow range, the step length of *α* variation can be taken to be a relatively large value for a fast search. The flow chart of the proposed rolling bearing fault diagnosis method is shown in Figure 1. The main steps are described as follows.

Step 1: The value range of *α* is set as [*α*_min_, *α*_max_], and its increasing step length is set as *s_α_*. Then, *α* increases step by step from *α*_min_, and in the *m*th step, the *α* value is expressed as *α_m_* = *α*_min_ + (*m* − 1)·*s_α_*.

Step 2: The SVMD algorithm is implemented with the corresponding parameter balancing *α_m_* in each step for the rolling bearing vibration signal to obtain a series of modes. The value of the EP index for each mode is calculated, and then the mode with the minimum EP value expressed as min*EP*(*m*) is selected as the target mode, whose order number is the *n*th mode expressed as *ON*(*m*).

Step 3: After *α* increases to *α*_max_ and the relevant calculation of the last step is completed, the *α_m_*–min*EP*(*m*) and *α_m_*–*ON*(*m*) curves are plotted. In the *α_m_*–min*EP*(*m*) curve, the *α* value corresponding to the minimum min*EP*(*m*) value is selected as the globally optimal *α* value and expressed as *α*_opt_.

Step 4: The SVMD algorithm is implemented with the balancing parameter value of *α*_opt_ for the rolling bearing vibration signal to obtain a series of modes, among which the optimal target mode is selected according to the order number at *α*_opt_ in the *α_m_*–*ON*(*m*) curve.

Step 5: SES analysis is performed for the optimal target mode to extract the FCF and diagnose the rolling bearing fault.

## 5. Simulation Analysis

In this section, a simulated vibration signal of a faulty rolling bearing is constructed to evaluate the efficiency of the proposed method. Considering a rolling bearing running with constant speed and assuming its inner race, outer race, or rollers have local damage, the excited vibration signal can be modeled as a series of periodic transient impulse features [38,39], and the vibration acceleration signal *x*(*t*) measured from the rolling bearing can be modeled as Equations (18)*–*(20):

(18) x(t)=s(t)+n(t)

(19) $$s(t)=\sum_{m=1}^{M}A_{m}e^{-\zeta (t-mT_{p}-\sum_{i=1}^{m}\tau_{i})}cos\left(2\pi f_{n}(t-mT_{p}-\sum_{i=1}^{m}\tau_{i})\right)\cdot u(t-mT_{p}-\sum_{i=1}^{m}\tau_{i})$$

(20) $$A_{m}=1+a_{m}\cdot \cos(2\pi f_{r}t)$$

where *s(t*) is an ideal impulsive vibration signal with no noise; *n*(*t*) is white Gaussian noise; *M* is the number of the fault impulses induced by the local damage; *A_m_* is the amplitude of the *m*th fault impulse; *a_m_* is the amplitude modulation coefficient, where 0 < *a_m_* < 1; *f*_r_ is the rotating frequency of the bearing; *ζ* is the structural damping coefficient; *T_p_* is the time period between two consecutive fault impulses, and *T_p_* = 1/*f*_c_, in which *f_c_* represents the FCF of inner race, outer race, or balls; *τ_i_* (*i* = 1, 2, …, *M*) represents the effect of random slippage of the balls and can be assumed to be a zero mean, uniformly distributed random sequence with a standard deviation of 0.01 *T_p_*~0.02 *T_p_*; *ω*_r_ is the excited resonance frequency; and *u*(*t*) represents the unit step function.

**Figure 2:**
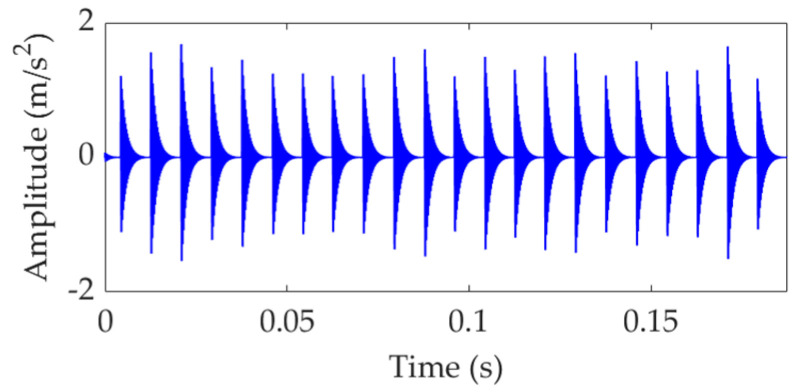
Vibration signal *s*(*t*) with fault-related impulsive features.

**Figure 3:**
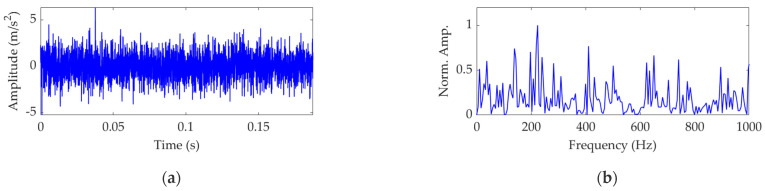
The simulated vibration signal *x*(*t*): (**a**) time−domain waveform and (**b**) SES.

The vibration signal of a faulty rolling bearing can be generated by setting the appropriate values for the relevant parameters. The parameters are set as *ζ* = 700 N.s/m, *f*_c_ = 120 Hz, *T_p_* = 0.0083 s, and *ω*_r_ = 8000π rad/s. The amplitude sequences *a_m_* (*m* = 1, 2, …, *M*) are obtained from a normal distribution with a mean of 0.5 and a standard deviation of 0.3. The standard deviation of the random sequences *τ_i_* (*i* = 1, 2, …, *M*) is set as 0.015 *T_p_*. The sampling frequency *f*_s_ is set as 16,000 Hz, and the number of sampling points is 3000. The vibration signal *s*(*t*) with ideal fault impulse features is shown in Figure 2. Then, *s*(*t*) is mixed with white Gaussian noise *n*(*t*) to achieve a simulated vibration signal *x*(*t*) with a signal-to-noise ratio (SNR) of −13 dB. The simulation vibration signal *x*(*t*) is shown in Figure 3a. The SNR of *x*(*t*) is so low that the fault-related impulsive features are completely overwhelmed by the noise and almost impossible to identify. The FCF is also unable to be extracted by SES, as shown in Figure 3b, in which the amplitudes of SES are normalized using the division-by-maximum method, and thus the ordinate represents the normalized amplitude, abbreviated as Norm. Amp.

To apply the SVMD algorithm to process the simulated vibration signal *x*(*t*), we first need to find a relatively optimal value for the balancing parameter *α* of SVMD. Subsequently, we let *α* increase gradually from a minimum value of 50 to a maximum value of 5000 at a step size of 50. In each step of *α* increase, *x*(*t*) is decomposed using the SVMD algorithm with the corresponding *α* value to obtain a series of modes, and the EP index value is calculated for each mode. One thing to note is that, in this simulated signal, only one FCF is involved, i.e., *f*_c_ = 120 Hz. The calculation of the PA index shown in Equation (15) should be accordingly modified as follows:

(21) $$PA=\left|f_{ma}-f_{c}\right|$$

**Figure 4:**
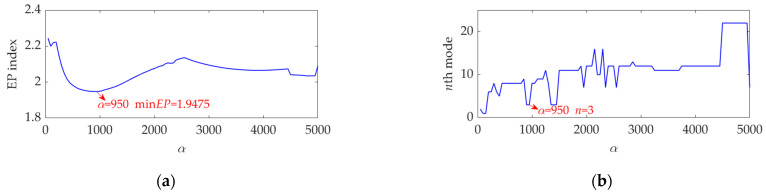
Relationship between the *α* value and (**a**) the EP index value and (**b**) order number of the target mode in each step of *α* increase.

Then, the mode with the minimum EP index value is selected as the target mode in the current step. After *α* increases to the maximum value and the last target mode is achieved, we can draw the relationship curve between the *α* value and the EP index value of the target mode in each step of *α* increase, which is shown in Figure 4a. At the same time, we can also draw the relationship curve between the *α* value and the order number of the target mode among the decomposed modes in each step, which is shown in Figure 4b.

Figure 4a shows that when the *α* value is 950, the EP index of the obtained target mode acquires a global minimum value, and thus the optimal value of *α* is achieved, expressed as *α*_opt_ = 950. Meanwhile, Figure 4b shows that, when the *α* value is 950, the target mode is the third mode. What we need to highlight is that, as the *α* value varies, both the total number of modes obtained by SVMD and the order number of the target mode are very likely to change accordingly. When the *α* value is large, there may be a large number of modes obtained due to narrow bandwidth, and it is always difficult to select the target mode. If the *α* value is set improperly, the selected target mode may not be a useful mode that can be used for the rolling bearing fault diagnosis. The existences of these problems reflect the necessity of this study, and the solutions of these problems represent the significance of this paper.

**Figure 5:**
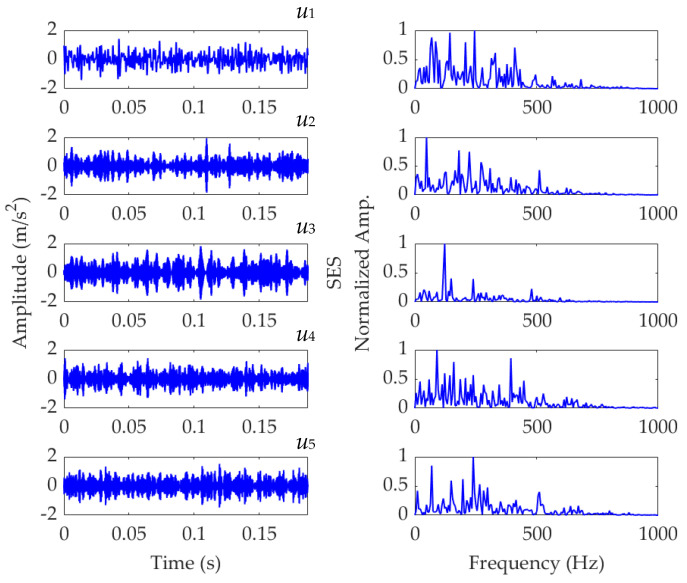
Results of the simulated vibration signal *x*(*t*) obtained using SVMD with *α*_opt_ = 950.

**Figure 6:**
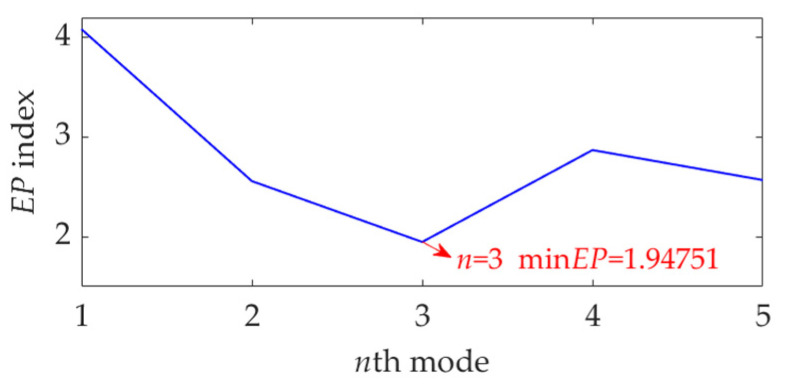
The EP index values of all modes.

**Figure 7:**
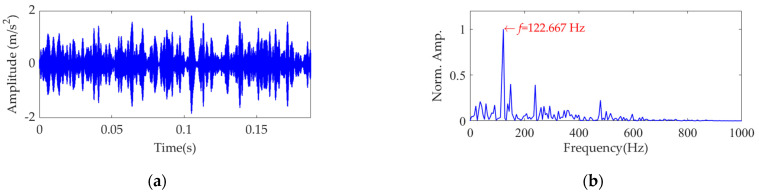
The optimal target mode: (**a**) time−domain waveform and (**b**) SES.

Now, we set the balancing parameter *α* of SVMD as *α*_opt_ = 950, and the decomposition results along with the corresponding squared envelope spectra of all modes are shown in Figure 5. The EP index values of all modes are shown in Figure 6. It can be seen that the total number of the modes is 5 and the third mode achieves the minimum value for the EP index, which is consistent with the result shown in Figure 4b. Therefore, the optimal target mode is the third mode, with details shown in Figure 7a, and its specific SES shown in Figure 7b. The extracted FCF is *f* = 122.667 Hz, which is basically consistent with the theoretical FCF of 120 Hz. The difference between the two values is caused by the effect of random slippage of the balls, considered in the simulated signal model, and the heavy noise. The process and results of the simulation analysis validate that the proposed EP index can effectively indicate the target mode from the results of SVMD, and the proposed method can successfully extract the simulated fault feature from the vibration signal with a low SNR, and thus be used for rolling bearing diagnosis.

**Figure 8:**
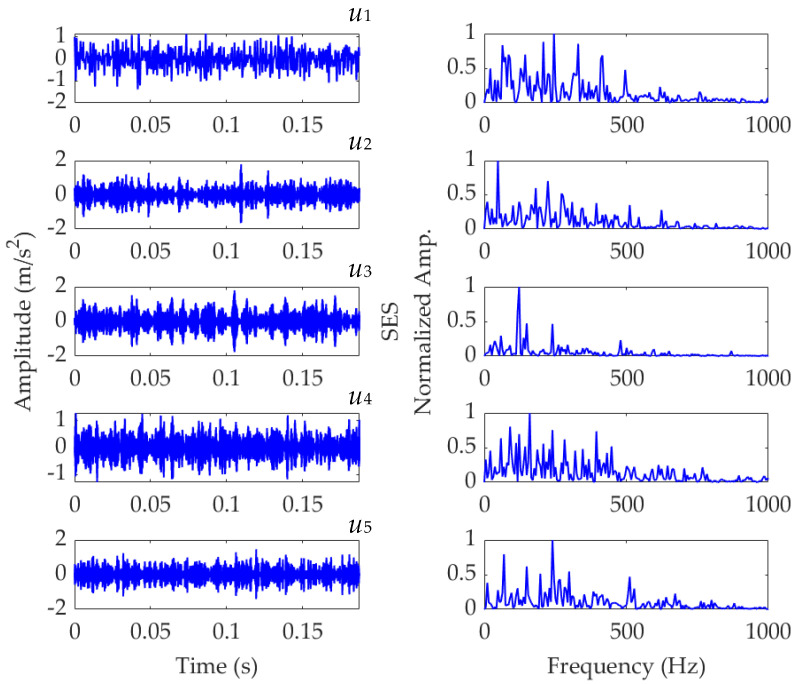
Results of the simulated vibration signal *x*(*t*) obtained by VMD.

**Figure 9:**
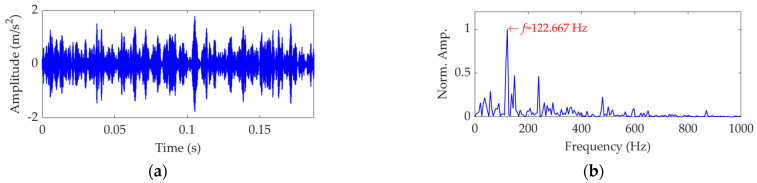
The target mode obtained by VMD: (**a**) time−domain waveform and (**b**) SES.

For comparison, the simulated vibration signal *x*(*t*) is processed using VMD. In the implementation of VMD, the number of modes is set as 5, and the balancing parameter *α* value is set as 950, which are the same as those described in the previous analysis of SVMD. The results obtained by VMD are shown in Figure 8. The target mode is also the third mode shown in Figure 9a, and its SES is shown in Figure 9b. On the premise of reasonable parameter settings, VMD can also successfully extract the simulated FCF of 122.667 Hz. As the FCFs extracted by SVMD and VMD have the same value, the PA indices of the corresponding target modes, calculated by Equation (15) or (21), achieve the same value. However, the EC index of the target mode obtained by SVMD is calculated to be 0.7264 according to Equation (14), while the EC index of the target mode obtained by VMD is calculated to be 0.7264. According to the definition of the EC index, the greater value of the EC index means that the fault characteristic component in the target mode is more prominent, and the degree of suppression of interference components with high energy is better. Therefore, in terms of the EC index, the performance of SVMD has certain superiority over VMD. Additionally, as VMD has two key parameters, i.e., the number of modes and balancing parameter *α*, needing to be set reasonably or optimized, while SVMD has only one, i.e., the balancing parameter *α*, the implementation of the SVMD method is more efficient than that of the VMD method under the same conditions.

## 6. Experimental Evaluation

In this section, the effectiveness of the proposed rolling bearing fault diagnosis method is investigated using three experimental vibration datasets. We focus on the fault feature extraction for the inner race, outer race, and balls of the rolling bearing. Based on this, we use the proposed method to extract the actual FCF from the vibration dataset of the rolling bearing and then compare the possible FCF with the theoretical FCFs of the three components mentioned above. The component whose FCF is closest to the actual FCF can be considered as the faulty component. This is the basic principle of the proposed method for rolling bearing fault diagnosis. According to the previous analysis, we express the theoretical FCFs of the inner race, outer race, and balls of the rolling bearing as *f*_ir_, *f*_or_, and *f*_ba_, respectively. Then, the PA index shown in Equation (15) needs to be specifically modified as Equation (22):

(22) $$PA={\left(\left|f_{\mathrm{ma}}-f_{\mathrm{ir}}\right|\cdot \left|f_{\mathrm{ma}}-f_{\mathrm{or}}\right|\cdot \left|f_{\mathrm{ma}}-f_{\mathrm{ba}}\right|\right)}^{1/3}$$

The calculation of the EP index should also be adjusted accordingly.

**Figure 10:**
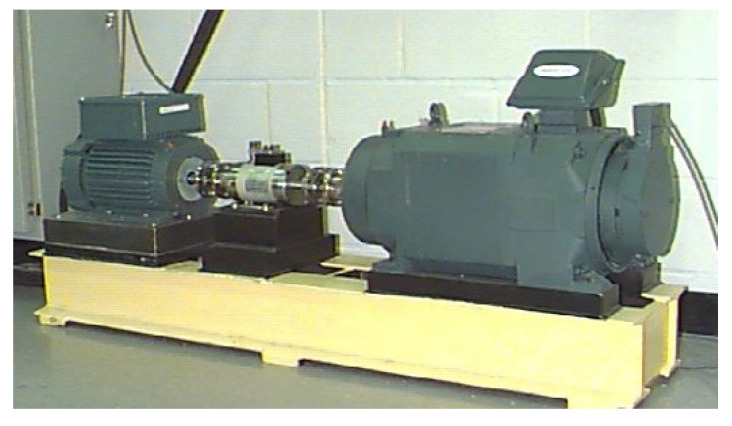
Rolling bearing testbed.

In the study of this paper, the experimental datasets associated with the rolling bearing come from the Bearing Data Center of Case Western Reserve University [40]. The bearing testbed is shown in Figure 10, which consists of a driving motor (left), a torque transducer/encoder (center), and a dynamometer (right). The driving motor shaft is supported by the test bearing, which was implanted with single point fault using electro-discharge machining. Vibration data associated with the test bearing were collected using accelerometers. Vibration signals were collected using a 16-channel DAT recorder and were post processed in a Matlab environment. The sampling frequency of vibration data was 12,000 Hz. Speed and horsepower data were collected using the encoder and torque transducer, respectively. All the datasets to be analyzed were selected as the vibration datasets of the drive end bearing.

### 6.1. Inner Race Fault-Related Vibration Dataset Analysis

In this case, we chose the test bearing with a single fault in the inner race, which was 0.007” in diameter and 0.011” in depth. The motor load was set as 3 HP. The actual speed of the motor was 1721 rpm, measured by the encoder, and thus the corresponding rotational frequency was *f*_r_ = 1721/60 = 28.6833 Hz. Then, the theoretical FCF of the inner race was calculated as *f*_ir_ = 5.4152·*f*_r_ = 155.3260 Hz, the theoretical FCF of the outer race was calculated as *f*_or_ = 3.5848·*f*_r_ = 102.8240 Hz, and the theoretical FCF of the ball was calculated as *f*_ba_ = 4.7135·*f*_r_ = 135.1989 Hz.

**Figure 11:**
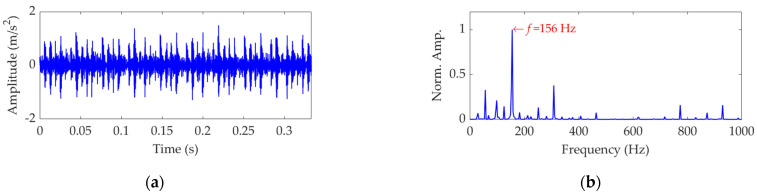
Vibration dataset associated with the inner race fault: (**a**) time−domain waveform and (**b**) SES.

The length of the sampled vibration data associated with the inner race fault was 122,917, and its mean value and standard deviation value were 0.0047 and 0.3136, respectively. A dataset selected from the sampled vibration data is shown in Figure 11a, and its mean value and standard deviation value are 0.0048 and 0.3128 respectively. The SES of the dataset is shown in Figure 11b, in which the actual extracted FCF is 156 Hz, consistent with the theoretical FCF of the inner race. Nevertheless, in order to verify the effectiveness of the proposed method, this vibration dataset was further processed using SVMD.

**Figure 12:**
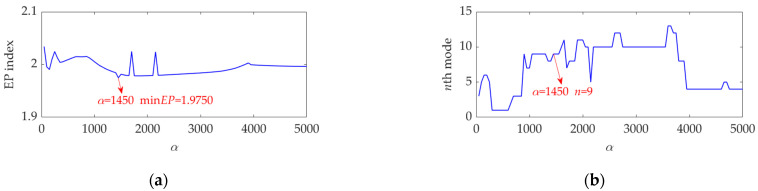
Relationship between the *α* value and (**a**) the EP index value and (**b**) order number of the target mode at each step of *α* increase.

**Figure 13:**
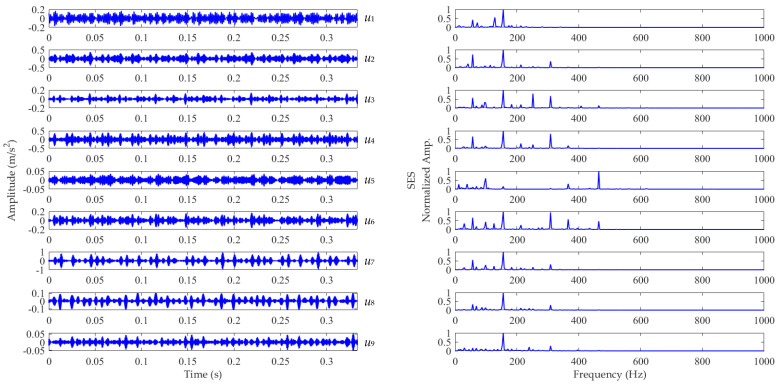
Results of the vibration dataset associated with the inner race fault obtained using SVMD with *α*_opt_ = 1450.

**Figure 14:**
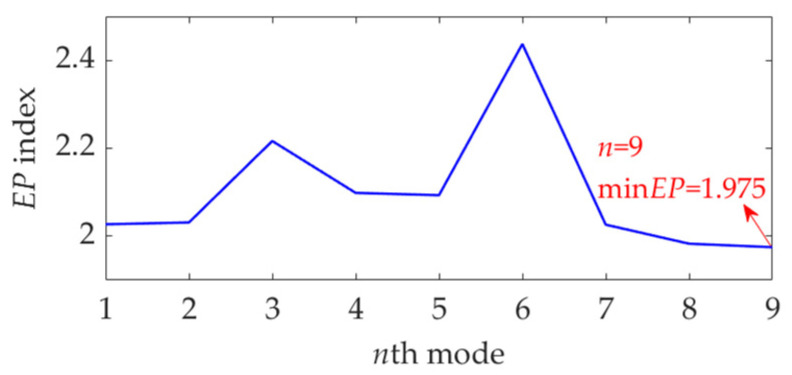
The EP index of each mode.

**Figure 15:**
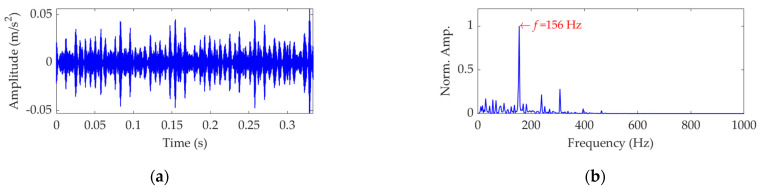
The optimal target mode: (**a**) time−domain waveform and (**b**) SES.

To find an optimal value for the balancing parameter *α* of SVMD, the *α* value was set to increase from 50 to 5000 at a step size of 50. In each step of *α* increase, SVMD was first implemented with the current *α* value for this vibration dataset to obtain a number of modes, then the EP index of each mode was calculated according to Equation (16), and finally the mode indicated by the minimum EP value was considered as the target mode corresponding to current step. When the *α* value increased to 5000 and the last target mode was achieved, the relationship curves between the *α* value and the EP index and order number of the target mode in each step were plotted, as shown in Figure 12. It can be seen that, when the *α* value is 1450, the EP index achieves an global minimum value, and the corresponding target mode is the ninth mode. Hence, we set the optimal value for the balancing parameter *α* of SVMD to be *α*_opt_ = 1450 and applied the SVMD algorithm to decompose the vibration dataset in this case. The decomposition results are shown in Figure 13. The variation curve of the EP index for each mode is shown in Figure 14. It can be seen that the ninth mode has the minimum value of the EP index, and it was thus chosen as the optimal target mode, which is consistent with the result in Figure 12b. The optimal target mode containing the complete fault information about the inner race is shown with details in Figure 15a, and its SES is shown in Figure 15b. It can be seen that the actual extracted FCF is 156 Hz, which closely matches the theoretical FCF of the inner race, i.e., 155.3260 Hz, and other frequency components acting as interferences are well suppressed. As noise interference is inevitable in the rolling bearing vibration signal, and the rolling bearing speed is impossible to keep strictly constant due to rolling surface damage, there inevitably exists a difference between the actual extracted FCF and theoretical FCF. However, this difference is very small and perfectly acceptable. The experimental result of this case verifies the effectiveness of the proposed EP index and SVMD method for the rolling bearing inner race fault diagnosis.

**Figure 16:**
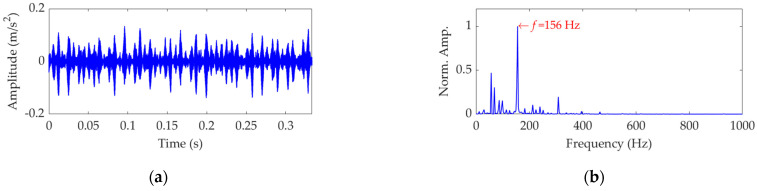
The target mode obtained by VMD: (**a**) time−domain waveform and (**b**) SES.

As a comparison, the vibration dataset of this case was decomposed using VMD. In the implementation of VMD, the number of modes was determined as 9 by experiments, and the balancing parameter *α* was optimally set as 500 using the line search method described previously. Among the modes obtained by the VMD method, the target mode was also the ninth mode, which is specifically shown in Figure 16a, and its SES is shown in Figure 16b. The FCF extracted by VMD is the same as that extracted by SVMD, which means that the PA indices of the target modes extracted by VMD and SVMD achieved the same value according to Equation (22). Nevertheless, the EC index of the target mode extracted by SVMD was calculated to be 0.8147 and was higher than that of the target mode extracted by VMD, which was calculated to be 0.7942. In this scenario, compared with the VMD method, the SVMD method is more effective in high energy interference components’ suppression.

### 6.2. Outer Race Fault-Related Vibration Dataset Analysis

In this case, the single implanted fault in the outer race of the test bearing was 0.021 in diameter and 0.011 in depth and located at six o’clock. The motor load was set as 3 HP. The measured motor speed was 1721 rpm using the encoder, and thus the corresponding rotational frequency was *f*_r_ = 1721/60 = 28.6833 Hz. As the test bearing in this case is the same as the previous case, the theoretical FCF of the inner race was *f*_ir_ = 155.3260 Hz, the theoretical FCF of the outer race was *f*_or_ = 102.8240 Hz, and the theoretical FCF of the ball was *f*_ba_ = 135.1989 Hz.

**Figure 17:**
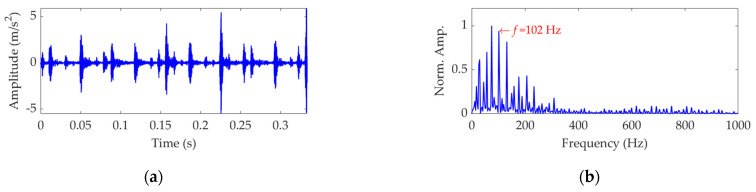
Vibration dataset associated with the outer race fault: (**a**) time−domain waveform and (**b**) SES.

The length of the sampled vibration data with regard to the outer race fault was 121,991 and its mean value and standard deviation value were 0.0035 and 0.5590, respectively. A dataset selected from the sampled vibration data is shown in Figure 17a, and its mean value and standard deviation value are 0.0030 and 0.5511, respectively. The SES of the dataset is shown in Figure 17b. Despite the significant impulsive features in Figure 17a, they do not show obvious periodicity and fail to relate with the FCF of the outer race. In addition, since the fault characteristic component indicated by the extracted actual FCF of 102 Hz is not dominant in the SES, and a lot of strong interference components exist, it is non-rigorous to assert that the outer race is faulty.

**Figure 18:**
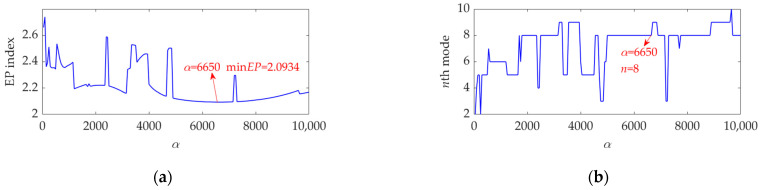
Relationship between the *α* value and (**a**) the EP index value and (**b**) order number of the target mode at each step of *α* increase.

**Figure 19:**
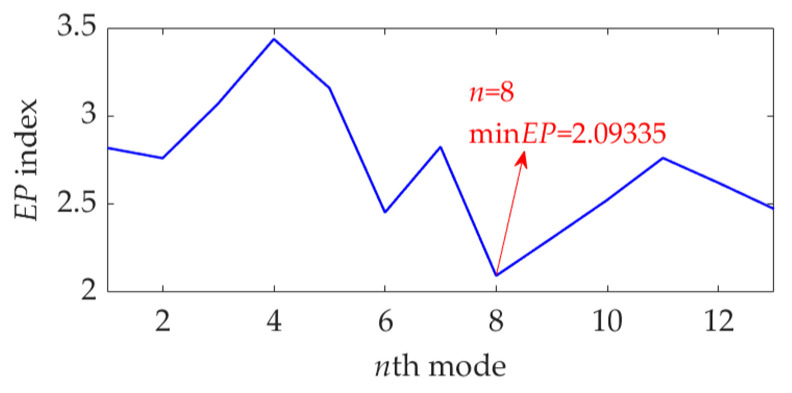
The EP index value of each mode.

**Figure 20:**
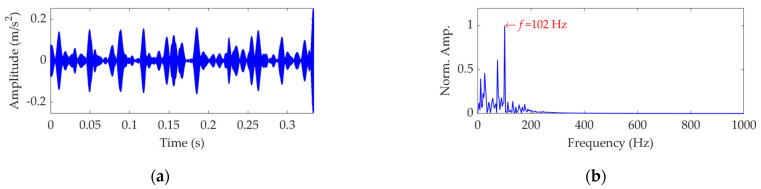
The optimal target mode: (**a**) time−domain waveform and (**b**) SES.

To apply the SVMD method to the dataset process of this case, the optimal value of the balancing parameter *α* was searched in the range 50–10,000, and then the *α* value increased from 50 to 10,000 at a step size of 50. The finally obtained relationship curves between the *α* value and the EP index and order number of the target mode in each step of *α* increase are shown in Figure 18. It can be seen that the optimal value of *α* is *α*_opt_ = 6650 because the corresponding target mode obtained by SVMD has the global minimum value for the EP index, and this optimal target mode is the eighth mode among the decomposition results. For more details, this dataset was decomposed using SVMD with the obtained optimal *α* value of 6650. The total number of the decomposed modes is 13, and the EP index of each mode is shown in Figure 19. The eighth mode with the minimum value of the EP index is the target mode, which is also consistent with the result indicated in Figure 18b. The target mode along with its SES is represented in Figure 20. From Figure 20b, we can see that as interference components are greatly removed or suppressed, the actual FCF of 102 Hz is extracted successfully, and the outer race is faulty, since the theoretical FCF of the outer race is 102.8240 Hz. Due to noise interference and very tiny variations of the bearing speed caused by the rolling surface damage, the difference between the actual extracted FCF and theoretical FCF is inevitable but perfectly acceptable. This result suggests that, with the help of the proposed EP index, the target mode can be well selected from the multiple modes obtained by SVMD, and the SVMD method with the optimized balance parameter can effectively extract the fault characteristics of outer race for the rolling bearing.

**Figure 21:**
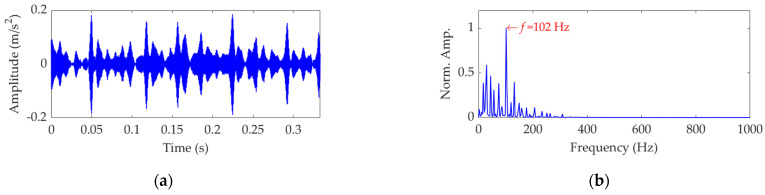
The target mode obtained by VMD: (**a**) time−domain waveform and (**b**) SES.

Now, we further use the VMD method to decompose the dataset in this case. According to the preceding analysis, we set the number of modes as 13 and the balancing parameter as 6650. In fact, this set of values is also a set of relatively optimal values for VMD, which has been validated by the linear search method mentioned previously. Among the results, the target mode is the fourth mode, as Figure 21a shows, and its SES is represented in Figure 21b. Although the VMD method is also able to extract the accurate FCF, in practice, it is cumbersome to acquire a set of relatively optimal values for the number of modes and balancing parameter of VMD. By comparison, the SVMD method is easier to implement. In addition, the EC index values of the target modes extracted by SVMD and VMD were calculated to be 0.6953 and 0.6406, respectively, which indicates that the target mode extracted by SVMD has lower energy interference components and, correspondingly, its fault characteristic component is more prominent.

### 6.3. Ball Fault-Related Vibration Dataset Analysis

In this case, one of the balls in the test bearing had a single implanted fault with 0.028” diameter and 0.011” depth. The motor load was set as 3 HP. The approximate speed of the motor was 1730 rpm, and the corresponding rotational frequency was *f*_r_ = 1730/60 = 28.83 Hz. Hence, the theoretical FCF of the inner race was calculated as *f*_ir_ = 5.4152·*f*_r_ = 156.1202 Hz, the theoretical FCF of the outer race was calculated as *f*_or_ = 3.5848·*f*_r_ = 103.3498 Hz, and the theoretical FCF of the ball was calculated as *f*_ba_ = 4.7135·*f*_r_ = 135.8902 Hz.

**Figure 22:**
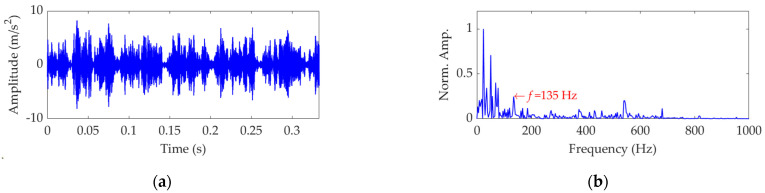
Vibration dataset associated with the ball fault: (**a**) time−domain waveform and (**b**) SES.

**Figure 23:**
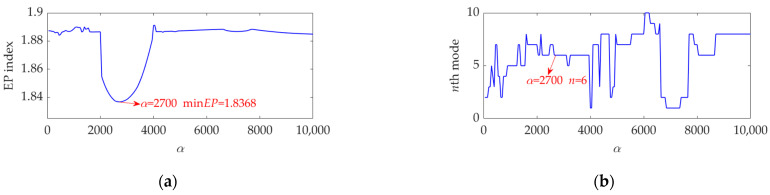
Relationship between the *α* value and (**a**) the EP index value and (**b**) order number of the target mode at each step of *α* increase.

**Figure 24:**
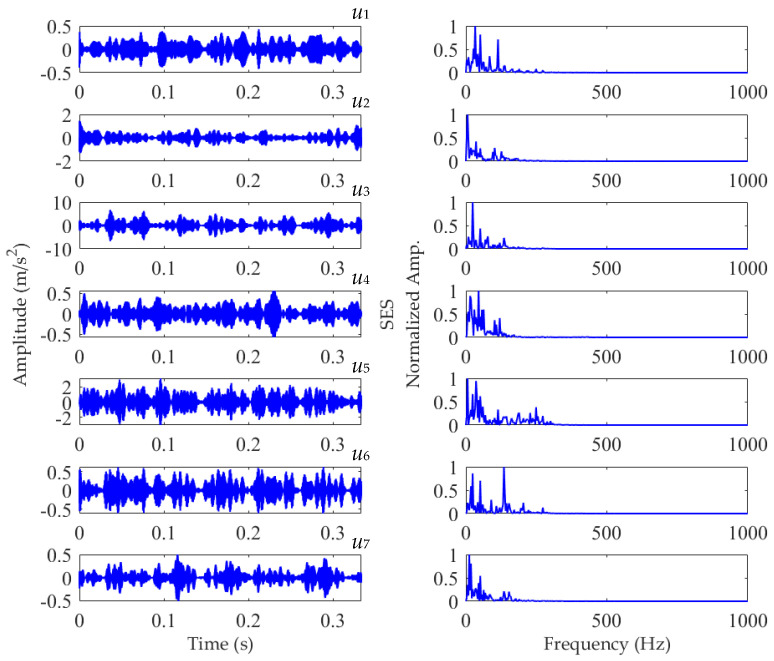
Results of the vibration dataset associated with the ball fault obtained using SVMD with *α*_opt_ = 2700.

**Figure 25:**
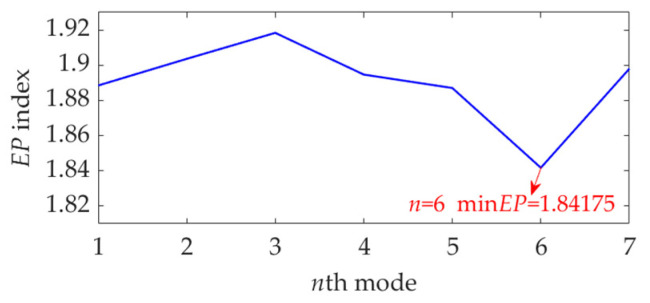
The EP index value of each mode.

**Figure 26:**
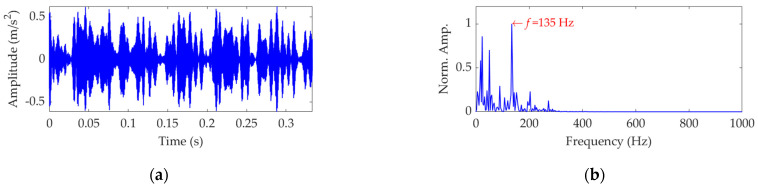
The optimal target mode: (**a**) time−domain waveform and (**b**) SES.

The length of the sampled vibration data corresponding to the ball fault was 120,984, and its mean value and standard deviation value were 0.0190 and 2.1449, respectively. A dataset selected from the sampled vibration data, together with its SES, is shown in Figure 22. The mean value and standard deviation value of the dataset were 0.0142 and 2.1156, respectively. As in the SES, the interference components were dominant and the fault characteristic component itself was quite weak; it is a challenging task to identify the correct FCF and judge that a ball in the rolling bearing is faulty. Hence, we further used the SVMD method to analyze this vibration dataset. To determine a proper value for the balancing parameter *α* of SVMD, the *α* value was increased from 50 to 10,000 at a step size of 50, and in each step, the target mode among the decomposed modes by SVMD was selected with the help of the EP index. Eventually, we could obtain the relationship curves between the *α* value and the EP index, as well as the order number of the target mode in each step of *α* increase, all of which are shown in Figure 23. It can be seen that when the *α* value is 2700, the target mode obtained by SVMD has the global minimum value for the EP index. Therefore, in this case, the optimal value for the balancing parameter *α* of SVMD is determined as *α*_opt_ = 2700, and the corresponding target mode is the sixth mode indicated in Figure 23b. Then, the vibration dataset of the faulty ball was decomposed by SVMD with the optimal *α* value of 2700 and the results are shown in Figure 24. The EP index values of all modes are displayed as Figure 25. Hence, the optimal target mode is the sixth mode with the minimum value of the EP index, which is in agreement with the result in Figure 23b. To be specific, the optimal target mode and its SES are shown in Figure 26. It can be seen that the fault characteristic component is the strongest, and the FCF of the ball, i.e., 135 Hz, can be easily identified, which is very close to the theoretical value of 135.8902 Hz. Considering the effect of random slippage of the balls, as well as the noise interference and the slight fluctuation of the bearing speed caused by rolling surface damage, there inevitably exists a difference between the actual FCF and theoretical FCF of the ball. Nevertheless, the actual extracted FCF is accurate enough and acceptable. Such a result shows that, guided by the proposed EP index, the SVMD method can effectively extract the useful fault characteristic information from rolling bearing vibration signal with strong interferences.

**Figure 27:**
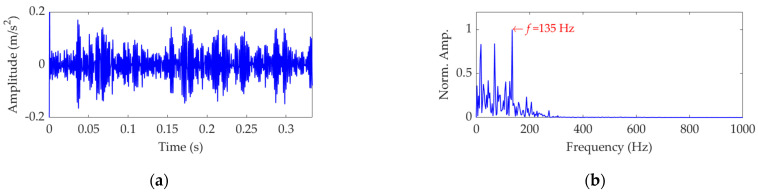
The target mode obtained by VMD: (**a**) time−domain waveform and (**b**) SES.

Lastly, the vibration dataset of this case was also decomposed by the VMD method for comparison. The balancing parameter *α* of VMD was set as the optimal value of 2700 used in the SVMD method. The number of modes was set as 20, which is an optimal value determined by multiple experiments. In the results obtained by VMD, the target mode was the 18th mode, whose waveform and SES are shown in Figure 27. It can be seen that, as long as the parameters are set properly, VMD is also able to extract the FCF of the rolling bearing ball from the vibration dataset. However, it is important to note that, in a practical application, the two key parameters of VMD are not easy to determine. As only one key parameter, i.e., the balancing parameter *α*, needs to be optimized in SVMD, the SVMD method shows higher efficiency than the VMD method under the same conditions. For further comparison, the EC indices of the target modes extracted by SVMD and VMD were calculated according to Equation (14) and achieved the values of 0.5702 and 0.4793, respectively. In this case, the target mode extracted by SVMD also has a better EC index and more prominent fault characteristic component.

## 7. Conclusions

In this paper, we propose a rolling bearing fault diagnosis method based on SVMD and the EP index. As there is only the balancing parameter needing to be optimized for SVMD, the SVMD method is more feasible to implement than the VMD method, which needs to optimize the number of modes and balancing parameter simultaneously. Nevertheless, it is not easy to determine the optimal value of the balancing parameter for SVMD, and the target mode containing ideal fault characteristic information is difficult to select from the multiple modes obtained by SVMD. In view of the existing shortcomings of SVMD, which are also true in VMD, the new proposed EP index can effectively indicate the target mode from the results of SVMD. Accordingly, an optimal value for the balancing parameter of SVMD can be easily achieved using the line search method guided by the EP index. The simulation and experimental results verify the effectiveness and practicability of the EP index and also demonstrate that the SVMD method has strong anti-noise and anti-interference ability, and thus can successfully extract the fault feature from vibration signal to realize the rolling bearing fault diagnosis. In addition, quantified by the new proposed EC index, the SVMD method shows better performance in interference suppression and fault feature enhancement than the VMD method. In the future, we may apply the SVMD method and EP index to fault feature extraction and fault diagnosis for a multistage gearbox, especially the wind turbine gearbox.

## Data Availability

Not applicable.
